# Grade III blunt splenic injury without contrast extravasation - World Society of Emergency Surgery Nijmegen consensus practice

**DOI:** 10.1186/s13017-020-00319-y

**Published:** 2020-08-03

**Authors:** Francesco Amico, Rebecca Anning, Cino Bendinelli, Zsolt J. Balogh, Participants of the 2019 World Society of Emergency Surgery (WSES) Nijmegen splenic injury collaboration group., Ari Leppäniemi, Daniel Aparicio-Sánchez, Erik Hermans, Federico Coccolini, Fikri M. Abu-Zidan, Massimo Chiarugi, Catherine Arvieux, Giovanni Pirozzolo, Vladimir Khokha, Matti Tolonen, Tan Edward, Michael Sugrue, Marco Ceresoli, Måns Muhrbeck, Rajashekar Mohan, Frank Piscioneri, Giuseppe Ietto, Osvaldo Chiara, Fausto Catena

**Affiliations:** 1 Department of Traumatology, John Hunter Hospital, Locked Bag 1, Hunter Region Mail Centre, Newcastle, NSW 2310 Australia; 2 School of Medicine and Public Health, University of Newcastle, Newcastle, NSW Australia; 3 Department of Surgery, John Hunter Hospital, Newcastle, NSW Australia; 4 Abdominal Center, Helsinki University Hospital, Meilahti, Finland; 5 Emergency Surgery Department in Virgen del Rocio University Hospital, Seville, Spain; 6 Radboud university medical center, Nijmegen, The Netherlands; 7 General, Trauma and Emergency Surgery Department, Pisa University Hospital, Pisa, Italy; 8 Department of Surgery, College of Medicine and Health Sciences, UAE University, Al-Ain, United Arab Emirates; 9 Emergency Surgery Unit & Trauma Center University of Pisa Cisanello Hospital, Pisa, Italy; 10 Department of Digestive and Emergency Surgery Grenoble Alpes University Hospital, Malakoff, France; 11 General Surgery Department Dell’Angelo Hospital, Venice, Italy; 12 City Hospital, Mozyr, Belarus; 13 Abdominal Center, Helsinki University Hospital and University of Helsinki, Helsinki, Finland; 14 Radboud university medical center, Nijmegen, The Netherlands; 15 Letterkenny University Hospital, Letterkenny, Ireland; 16 Department of general and emergency surgery, University of Milano-Bicocca, Monza, Italy; 17 Department of Surgery in Norrköping, Center for Surgery, Orthopaedics and Cancer Treatment, County Council Östergötland, Norrköping, Sweden; 18 Division of Surgery, Department of Biomedical and Clinical Sciences, Faculty of Health Sciences, Linköping University, Norrköping, Sweden; 19 Department of Surgery, K.S. Hegde Medical Academy of NITTE, Mangalore, India; 20 The Canberra Hospital, Canberra, Australia; 21 General, Emergency and Transplant Surgery Department, University of Insubria, Varese, Italy; 22 Trauma and Acute Care Surgery, ASST Niguarda Hospital, Milano, Italy; 23 Emergency Surgery dept., Parma University Hospital, Parma, Italy

**Keywords:** Trauma, Spleen, Injury, Blunt, Grade III, WSES, Consensus, Questionnaire, Practice variation

## Abstract

**Background:**

Recent trauma guidelines recommend non-operative management for grade III splenic injury without contrast extravasation on computed tomography. Nevertheless, such recommendations rely on low-quality evidence, and practice variation characterizes clinical management for this type of injury. We aimed to identify the role of eleven selected clinical factors influencing the management of grade III splenic injury without contrast extravasation by expert consensus and a modified Delphi approach.

**Methods:**

A questionnaire was developed with the endorsement of the World Society of Emergency Surgery (WSES). This was delivered and answered live by acute care surgeons attending the 6^th^ WSES congress in Nijmegen in 2019. A dedicated mobile phone application was utilized to collect the answers. All answers were evaluated for areas of discrepancy with an 80% threshold for consensus between respondents.

**Results:**

Three factors generated discrepancy in opinion for managing this pattern of injury: the patients’ injury severity, the presence of a bleeding diathesis, and an associated intra-abdominal injury. Agreement was obtained for the other eight factors.

**Conclusion:**

Researchers should focus their efforts on the identified area of discrepancy. Clinicians should use additional care in the presence of the three factors for which discordant opinions were found.

## Background

The spleen is the most commonly injured solid organ in blunt abdominal trauma and contributes to worldwide trauma associated mortality and morbidity [1]. Over the last 30 thirty years, there has been a prominent shift towards a more conservative approach in the management of splenic injury, with an emphasis on the preservation of splenic parenchyma and function [2]. Indeed, current data suggests up to 90% of patients with splenic injury can be treated non-operatively, boasting a success rate of over 80% in avoiding surgical intervention [3]. This shift was aided by haemostatic resuscitation, enhanced diagnostic, monitoring facilities and advances in the field of interventional radiology with selective or non-selective splenic artery angioembolisation [4]. As a result, patients have benefited from lower mortality rates, shorter hospital stays and decreased burden of post-splenectomy complications.

High level evidence for management of splenic injury is limited [5], and guidelines necessarily rely on studies with less than optimal design [6]. The American Association for the Surgery of Trauma (AAST) grading (herein referred upon as grade) and the presence of contrast extravasation on computed tomography (CT) (henceforth referred upon as blush) play an important role in planning the management of splenic injuries. While the management of splenic injury with blush [7] and/or grade IV-V splenic injuries [8] is supported by studies on large databases, the management of grade III injury without blush is not supported by large cohort studies. As a result, previous attempts to gain consensus on the management of this specific injury pattern have consistently failed [9]. The World Society of Emergency Surgery (WSES) guidelines indicate such injury not warranting angiography/angioembolisation [6]. Nevertheless, a high rate of practice variation hinders management of grade III splenic lesions [10]. This can be also confounded by factors like patient age, associated injury, presence of haemoperitoneum, co-morbidities and overall injury severity [11].

We hypothesized that experienced clinicians would meet consensus on areas of clinical variation regarding the management of grade III blunt splenic injuries without CT blush when presented with a clinically relevant hypothetical scenario. A modified Delphi questionnaire was utilised in the form of a phone application, with the aim to obtain expert opinion and enhance effective decision-making in the management of grade III blunt splenic injury without blush.

## Methods

**Fig. 1:**
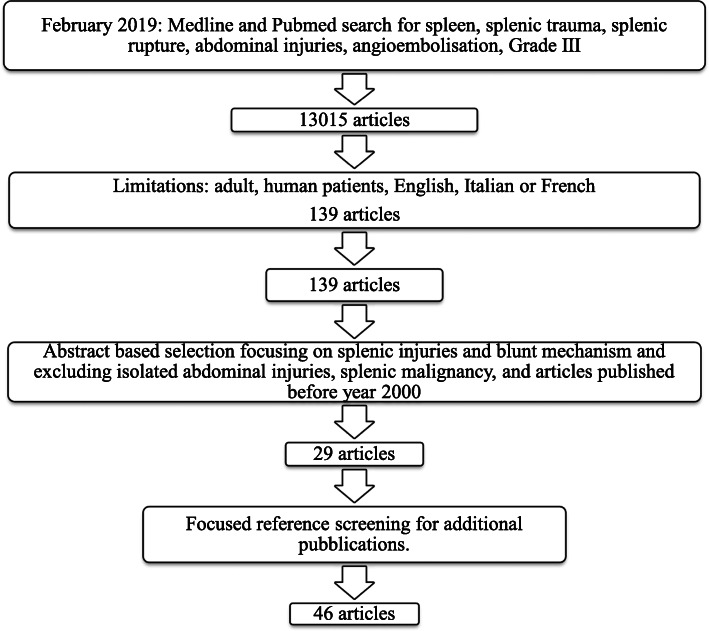
Literature review

Factors influencing the management of grade III splenic injures without blush were identified in order to incorporate debatable key topics in this work. For this purpose, in February 2019, we performed a PubMed and Medline database literature search for articles published since 2000 in English, Italian and French. The terms “spleen”, “splenic trauma”, “splenic rupture”, “abdominal injuries” “angioembolisation” and “grade III” were searched. One hundred and thirty-nine articles were found. Following subsequent abstract-based paper selection and focused reference screening for additional relevant publications, 46 articles were identified to assist in the creation of the questionnaire (Fig. 1) (Appendix).

**Fig. 2:**
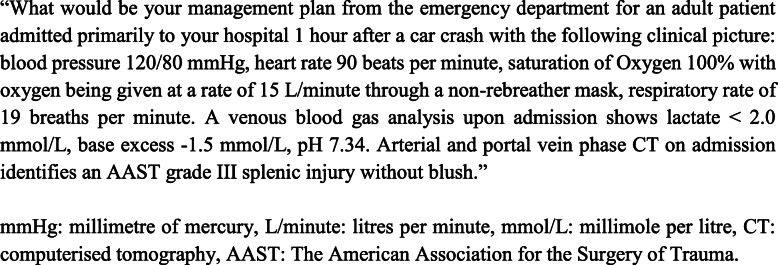
Clinical scenario. “What would be your management plan from the emergency department for an adult patient admitted primarily to your hospital 1 hour after a car crash with the following clinical picture: blood pressure 120/80 mmHg, heart rate 90 beats per minute, saturation of Oxygen 100% with oxygen being given at a rate of 15 L/minute through a non-rebreather mask, respiratory rate of 19 breaths per minute. A venous blood gas analysis upon admission shows lactate <2.0 mmol/L, base excess -1.5 mmol/L, pH 7.34. Arterial and portal vein phase CT on admission identifies an AAST grade III splenic injury without blush”. mmHg, millimetre of mercury; L/min, litres per min; mmol/L, millimole per litre; CT, computerised tomography; AAST, The American Association for the Surgery of Trauma

A clinically relevant hypothetical patient scenario was crafted for clinicians to formulate optimal management plans. Blood pressure, heart rate, oxygen saturation, supplemental oxygenation, venous lactate, base excess, pH and haemoglobin were provided (Fig. 2). Questions were endorsed by the WSES and designed to reflect real world practice. Clinicians were required to select their preferred course of action.

Each component of the questionnaire targeted an area of potential practice variation around eleven key patient and injury-related factors. Seven questions were related to injury variables and four to patient variables (Table 1). Experts were asked if each variable independently influenced their management. The option was given to answer “no”—with the patient receiving standard non-operative management or “yes”—with the option to favour angiography/angioembolisation or operative management.

**Table 1:** Questions on areas of practice variation

**Injury-related factors**	
1	Does the presence of a peri-splenic haemoperitoneum alone influence your management plan?
2	Does the presence of a head injury alone influence your management plan?
3	Does your perception of a worsening overall injury severity alone influence your management plan?
4	Does the presence of associated intra-abdominal injury alone influence your management plan?
5	Does the presence of associated extra-abdominal injury alone influence your management plan?
6	Does the initial need for administration of intravenous fluid replacement to achieve normal haemodynamic status alone influence your management plan?
7	Does an increasing time from injury alone influence your management plan?
**Patients related factors**	
8	Does an increasing age alone influence your management plan?
9	Does a history of previous left upper quadrant abdominal surgery alone influence your management plan?
10	Does the presence of a worsening comorbidity status alone influence your management plan?
11	Does the presence of a congenital or acquired non-reversible bleeding diathesis alone influence your management plan?

The questionnaire was completed by trauma and acute care surgery experts who convened to the 6th WSES congress in Nijmegen, The Netherlands, in June 2019. Participants were invited to respond live utilising a mobile phone application to facilitate response. The method solicited the opinions of experts, which were anonymously collated for analysis. Answers were examined for areas of discrepancy. Consensus was defined as an agreement of 80% amongst respondents (total of same answers divided by the number of respondents) [12].

## Results

There were fifty-three respondents. There were three questions that demonstrated a significant discrepancy: the presence of several other injuries, the presence of associated intra-abdominal injury and the presence of a non-reversible bleeding diathesis. Experts did not agree and did not reach the stipulated consensus level of 80% when asked if any of these three variables influenced their management plan. Consensus agreement was instead obtained for the other variables, with respondents indicating non-operative management as the preferred management option (Table 2).

**Table 2:** Collated answers

Questions		n (%)		
		No-NOM	Yes-AG/AE	Yes-OM
1	Peri-splenic haemoperitoneum	45 (90)	4 (8)	1 (2)
2	Head injury	43 (88)	3 (6)	3 (6)
3	Multiple injuries	17 (32)	21 (40)	15 (28)
4	Associated intra-abdominal injury	30 (62)	5 (10)	14 (28)
5	Associated extra-abdominal injury	42 (87)	2 (5)	4 (8)
6	Intravenous fluid replacement	41 (82)	4 (8)	5 (10)
7	Increasing time from injury	45 (95)	0	2 (5)
8	Age	41 (85)	1 (3)	6 (12)
9	Previous left upper quadrant surgery	46 (95)	1 (2)	1 (2)
10	Multiple comorbidities	42 (88)	5 (10)	1 (2)
11	Bleeding diathesis	22 (46)	17 (35)	9 (18)

*NOM* non-operative management, *AG/AE* angiography/angioembolisation, *OM* operative management

## Discussion

We observed broad agreement amongst WSES members. Experts confirmed their preference for a non-operative approach in response to most presented clinical variables, in keeping with previously published WSES guidelines [6]. Deviation from optimal care is a concern especially for grade III injury without blush, and an improvement in the management of this injury can result in higher splenic salvage rates and lower health care costs.

The following areas of agreement were identified amongst patient-related variables: older age, comorbidity status and previous surgery within the left upper quadrant of the abdomen. The audience agreed that non-operative management was indicated in the presence of these variables. Notwithstanding, the correlation between failure of non-operative management and age is noted within existing literature [11]. With regards to injury-related variables, non-operative management was the respondents’ preferred choice for patients with head injury, extra-abdominal injury, increased time of assessment from initial injury, presence of blood within the peri-splenic space or along the left paracolic gutter in proximity of the spleen and requirement of intravenous therapy to normalise haemodynamic status. The audience’s position on the latter of these aspects is supported in the literature [6]. Haemodynamic instability normally corresponds to intravenous fluid requirement and has in fact proved non-significant as predictor of non-operative management failure in a recent publication by Smith et al. [13].

The three areas of disagreement represent an interesting result of this work. Although some authors report a direct correlation between the overall injury severity and non-operative management failure, other groups showed different findings [11, 13]. This ambiguity is reflected in the answers we collected, with no agreement reached on the role of multiple injuries. It is noteworthy that there may be a potential source of bias from respondents driven by the difference between the perceived injury severity in the emergency department and a final calculated Injury Severity Score. This may relate to delays between time of finalising clinical assessment in the emergency department and time of imaging, or relate to the loss in discrimination power of ISS scores greater than 15 [14]. The question on the presence of a congenital or acquired non-reversible bleeding diathesis was another area which failed to reach consensus. This finding could be attributed to ill-defined factors linked to that question, for example, aetiology of the bleeding diastasis. A potential concern related to patient comorbidities is also possible. While multiple therapeutic options exist, coagulopathy is known to negatively impact the outcomes of patients with splenic injury [15]and is therefore worthwhile investigating in future research. The third area of disagreement is related to the presence of associated intra-abdominal injury. On this regard, the presence of concurrent solid organ injury has been found to have a significant correlation with prolonged admission and intensive care length of stay [16]. In review, this question may have been better phrased using the expression “solid organs” to allow for enhanced understanding amongst respondents avoiding potential confusion with intra-abdominal hollow viscus injury.

Targeted medical audiences have been surveyed for years with paper-based telephone and mail administered questionnaires [17]. A more modern internet and application-based approach has streamlined participants’ interrogation, but high response rates remain a problem. A reward-based approach in online surveys could help overcome that challenge. However, interference from the stakeholders could limit the results. A Cochrane review of fourteen studies regarding the use of applications in public health and clinical research has determined that their use may be equivalent to other delivery modes such as paper or email. Indeed, it found that responses were generally achieved faster, and data was more complete with a perhaps greater level of adherence to sampling than comparative paper models [18]. In the present study, experts from around the world gathered in one location; therefore, the response was immediate. Bringing the respondent to the survey instead of delivering the questionnaire to the recipient’s inbox might represent a better approach to this study methodology. Mobile phone-based questionnaires delivered to the audience of professional society meetings might provide an opportunity to maximise response rate, with minimal logistical effort and immediate turnaround time.

Blinded respondents and anonymous analysis are also strengths of the proposed approach. Also, this approach allows to comment on some specific aspects that might benefit from clarification in real time (i.e., role of sub-capsular haematoma). One limitation of this study is the performance of this questionnaire as a single round study, with answers not adjusted by respondents’ geographical origin or level of expertise. Answers were also burdened by a discrepant number of respondents, potentially due to presentation format and the time restraints. Additionally, the presentation and questionnaire were undertaken in English. Given the international setting, this may have posed a barrier for some non-English speaking respondents. Furthermore, potential bias from cumulative effect is a limitation to this approach that still provides low quality evidence, and phrasing is a well understood obstacle in any questionnaire-based research

## Conclusions

The present study indicated some discrepancy in the management of grade III blunt splenic injury without contrast extravasation among expert physicians, namely, splenic injury in the context of polytrauma with high extra-abdominal injury severity, congenital or acquired bleeding diathesis and associated intra-abdominal injury. These findings were obtained through real-time assessment tool of clinical practice of experts in a scientific meeting. This study could help guide future research pertaining to splenic injury.

## Data Availability

Available upon request to the corresponding author.

## Appendix

### Appendix. Articles selected to focus the questionnaire on areas of practice variation

**Table:**

1	Alabbasi T, Nathens A, Tien H. Blunt splenic injury and severe brain injury: a decision analysis and implications for care. Canadian Journal of Surgery. 2015;58(3):S108-S117.
2	Baygeldi S, Karakose O, Özcelik K, Pülat H, Damar S, Eken H et al. Factors Affecting Morbidity in Solid Organ Injuries. Disease Markers. 2016;2016:1-6.
3	Benjamin E, Cho J, Recinos G, Dilektasli E, Lam L, Brunner J et al. Negative computed tomography can safely rule out clinically significant intra-abdominal injury in the asymptomatic patient after blunt trauma. Journal of Trauma and Acute Care Surgery. 2018;84(1):128-132.
4	Beuran M, Gheju I, Venter MD, Marian RC, Smarandache R. Non-operative management of splenic trauma. J Med Life. 2012;5(1):47-58.
5	Bhullar I, Frykberg E, Siragusa D, Chesire D, Paul J, Tepas J et al. Age Does Not Affect Outcomes of Nonoperative Management of Blunt Splenic Trauma. Journal of the American College of Surgeons. 2012;214(6):958-964.
6	Bhullar I, Frykberg E, Siragusa D, Chesire D, Paul J, Tepas J et al. Selective angiographic embolization of blunt splenic traumatic injuries in adults decreases failure rate of nonoperative management. The Journal of Trauma and Acute Care Surgery. 2012;72(5):1127-1134.
7	Bhullar IS, Fryberg E, Tepas JJ, Siragusa D, Loper T, Kerwin AJ. At first blush: absence of computed tomography contrast extravasation in Grade IV or V adult blunt splenic trauma should not be preclude angioembolization. J Trauma Acute Care Surg. 2013;74(1):105-111.
8	Bhullar I, Tepas J, Siragusa D, Loper T, Kerwin A, Frykberg E. To nearly come full circle: Nonoperative management of high-grade IV–V blunt splenic trauma is safe using a protocol with routine angioembolization. Journal of Trauma and Acute Care Surgery. 2017;82(4):657-664.
9	Brault-Noble G, Charbit J, Chardon P, Barral L, Guillon F, Taourel P et al. Age should be considered in the decision making of prophylactic splenic angioembolization in nonoperative management of blunt splenic trauma. Journal of Trauma and Acute Care Surgery. 2012;73(5):1213-1220.
10	Brillantino A, Iacobellis F, Robustelli U, Villamaina E, Maglione F, Colletti O et al. Non operative management of blunt splenic trauma: a prospective evaluation of a standardized treatment protocol. European Journal of Trauma and Emergency Surgery. 2015;42(5):593-598.
11	Carr J, Roiter C, Alzuhaili A. Correlation of operative and pathological injury grade with computed tomographic grade in the failed nonoperative management of blunt splenic trauma. European Journal of Trauma and Emergency Surgery. 2012;38(4):433-438.
12	Chastang L, Bège T, Prudhomme M, et al. Is non-operative management of severe blunt splenic injury safer than embolization or surgery? Results from a French prospective multicenter study. J Visc Surg. 2015;152(2):85–91.
13	Cimbanassi S, Chiara O, Leppaniemi A, Henry S, Scalea TM, Shanmuganathan K, et al. Nonoperative management of abdominal solid-organ injuries following blunt trauma in adults: Results from an International Consensus Conference. J Trauma Acute Care Surg. 2018;84(3):517-31.
14	Coccolini F, Montori G, Catena F, Kluger Y, Biffl W, Moore EE, et al. Splenic trauma: WSES classification and guidelines for adult and pediatric patients. World Journal of Emergency Surgery. 2017;12(1):40.
15	Demetriades D, Hadjizacharia P, Constantinou C, Brown C, Inaba K, Rhee P et al. Selective Nonoperative Management of Penetrating Abdominal Solid Organ Injuries. Transactions of the Meeting of the American Surgical Association. 2006;124:285-293.
16	Demetriades D, Scalea T, Degiannis E, Barmparas G, Konstantinidis A, Massahis J et al. Blunt splenic trauma. The Journal of Trauma and Acute Care Surgery. 2012;72(1):229-234.
17	Duchesne J, Simmons J, Schmieg R, McSwain N, Bellows C. Proximal Splenic Angioembolization Does Not Improve Outcomes in Treating Blunt Splenic Injuries Compared With Splenectomy: A Cohort Analysis. The Journal of Trauma: Injury, Infection, and Critical Care. 2008;65(6):1346-1353.
18	Ermolov A, Tlibekova M, Yartsev P, Levitsky V, Chernysh O. Laparoscopic Splenectomy in Patients With Spleen Injuries. Surg Laparosc Endosc Percutan Tech. 2015;25(6):483-486.
19	Fata P, Robinson L, Fakhry SM. A survey of EAST member practices in blunt splenic injury: a description of current trends and opportunities for improvement. J Trauma. 2005;59(4):836–842.
20	Gardiner G. Nonoperative Management of Traumatic Splenic Injuries: Is There a Role for Proximal Splenic Artery Embolization?. Yearbook of Diagnostic Radiology. 2007:250-252.
21	Hsieh T, Tsai T, Liu Y, Hsieh C. How Does the Severity of Injury Vary between Motorcycle and Automobile Accident Victims Who Sustain High-Grade Blunt Hepatic and/or Splenic Injuries? Results of a Retrospective Analysis. International Journal of Environmental Research and Public Health. 2016;13(7):739.
22	Jabbour G, Al-Hassani A, El-Menyar A, Abdelrahman H, Peralta R, Ellabib M et al. Clinical and Radiological Presentations and Management of Blunt Splenic Trauma: A Single Tertiary Hospital Experience. Medical Science Monitor. 2017;23:3383-3392.
23	Jeremitsky E, Kao A, Chad C, Rodriguez A, Ong A. Does splenic embolization and grade of splenic injury impact nonoperative management of patients sustaining blunt splenic trauma?. The American Surgeon. 2011;77(2):215-220.
24	Karthikeyan S. To Operate or not to Operate, That is the Question! Review of Management of Splenic Trauma in our Institution over the Past 15 Years. New Indian Journal of Surgery. 2016;7(2):139-143.
25	Miller P, Chang M, Hoth J, Mowery N, Hildreth A, Martin R et al. Prospective Trial of Angiography and Embolization for All Grade III to V Blunt Splenic Injuries: Nonoperative Management Success Rate Is Significantly Improved. Journal of the American College of Surgeons. 2014;218(4):644-648.
26	Mohseni S, Holzmacher J, Sjolin G, Ahl R, Sarani B. Outcomes after resection versus non-resection management of penetrating grade III and IV pancreatic injury: A trauma quality improvement (TQIP) databank analysis. Injury. 2018;49(1):27-32.
27	Muroya T, Ogura H, Shimizu K, Tasaki O, Kuwagata Y, Fuse T et al. Delayed formation of splenic pseudoaneurysm following nonoperative management in blunt splenic injury. Journal of Trauma and Acute Care Surgery. 2013;75(3):417-420.
28	Olthof DC, Joosse P, van der Vlies CH, de Haan RJ, Goslings JC. Prognostic factors for failure of nonoperative management in adults with blunt splenic injury: a systematic review. J Trauma Acute Care Surg. 2013;74(2):546-57.
29	Olthof DC, Luitse JS, de Rooij PP, Leenen LP, Wendt KW, Bloemers FW, et al. Variation in treatment of blunt splenic injury in Dutch academic trauma centers. J Surg Res. 2015;194(1):233-8.
30	Olthof DC, van der Vlies CH, Goslings JC. Evidence-Based Management and Controversies in Blunt Splenic Trauma. Curr Trauma Rep. 2017;3(1):32-7.
31	Omert L, Salyer D, Dunham C, Porter J, Silva A, Protetch J. Implications of the “Contrast Blush” Finding on Computed Tomographic Scan of the Spleen in Trauma. The Journal of Trauma: Injury, Infection, and Critical Care. 2001;51(2):272-278.
32	Peitzman A, Ferrada P, Puyana J. Nonoperative Management of Blunt Abdominal Trauma: Have We Gone Too Far?. Surgical Infections. 2009;10(5):427-433.
33	Rosati C, Ata A, Siskin G, Megna D, Bonville D, Stain S. Management of splenic trauma: a single institution’s 8-year experience. The American Journal of Surgery. 2015;209(2):308-314.
34	Sabe AA, Claridge JA, Rosenblum DI, Lie K, Malangoni MA. The effects of splenic artery embolization on nonoperative management of blunt splenic injury: a 16-year experience. J Trauma. 2009;67(3):565–572.
35	Saurabh G, Kumar S, Gupta A, Mishra B, Sagar S, Singhal M et al. Splenic trauma - our experience at a level I Trauma Center. Turkish Journal of Trauma and Emergency Surgery. 2011;17(3):238-242.
36	Savage SA, Zarzaur BL, Magnotti LJ, Weinberg JA, Maish GO, Bee TK, et al. The evolution of blunt splenic injury: resolution and progression. The Journal of trauma. 2008;64(4):1085-92
37	Schnüriger B, Kilz J, Inderbitzin D, Schafer M, Kickuth R, Luginbühl M et al. The accuracy of FAST in relation to grade of solid organ injuries: A retrospective analysis of 226 trauma patients with liver or splenic lesion. BMC Medical Imaging. 2009;9(1).
38	Shamim S, Razzak J, Umer S, Chawla T. Splenic Injury After Blunt Abdominal Trauma: An Unusual Presentation. The Journal of Emergency Medicine. 2011;41(5):489-491.
39	Smalls N, Obirieze A, Ehanire I. The impact of coagulopathy on traumatic splenic injuries. American journal of surgery. 2015;210(4):724-9.
40	Smith S, Morris L, Spreadborough S, Al-Obaydi W, D’Auria M, White H et al. Management of blunt splenic injury in a UK major trauma centre and predicting the failure of non-operative management: a retrospective, cross-sectional study. European Journal of Trauma and Emergency Surgery. 2017;44(3):397-406.
41	Stassen NA, Bhullar I, Cheng JD, et al. Selective nonoperative management of blunt splenic injury: an Eastern Association for the Surgery of Trauma practice management guideline. J Trauma Acute Care Surg. 2012;73(5 Suppl 4): S294–S300.
42	Tan K, Chiu M, Vijayan A. Management of isolated splenic injuries after blunt trauma: an institution's experience over 6 years. Medical Journal of Malaysia. 2010;65(4):304-6
43	Teuben MPJ, Spijkerman R, Blokhuis TJ, Pfeifer R, Teuber H, Pape HC, et al. Safety of selective nonoperative management for blunt splenic trauma: the impact of concomitant injuries. Patient safety in surgery. 2018; 12:32.
44	Zarzaur BL, Kozar RA, Fabian TC, Coimbra R. A Survey of American Association for the Surgery of Trauma Member Practices in the Management of Blunt Splenic Injury. Journal of Trauma and Acute Care Surgery. 2011;70(5):1026-31.
45	Zarzaur BL, Croce MA, Fabian TC. Variation in the use of urgent splenectomy after blunt splenic injury in adults. J Trauma. 2011;71(5):1333–1339.
46	Zarzaur B, Rozycki G. An update on nonoperative management of the spleen in adults. Trauma Surgery & Acute Care Open. 2017;2(1).

## Abbreviations

WSES: World Society of Emergency Surgery
AAST: The American Association for the Surgery of Trauma
CT: Computerised tomography
